# Editorial: Insight in geriatric pain–2023

**DOI:** 10.3389/fpain.2025.1690268

**Published:** 2025-09-17

**Authors:** Kelly Marie Naugle, Keela Herr

**Affiliations:** ^1^ Department of Kinesiology, School of Health and Human Sciences, Indiana University Indianapolis, Indianapolis, IN, United States; ^2^ College of Nursing, University of Iowa, Iowa City, IA, United States

**Keywords:** geriatric pain, older adults, chronic pain, frailty, pain management

**Editorial on the Research Topic**
Insight in geriatric pain–2023

This editorial presents a series of articles from the research topic “Insights in Geriatric Pain” published in the *Frontiers in Pain Research Journal* – Geriatric Pain section. Chronic pain is the leading cause of disability (1, 2), facilitates functional decline and sedentary behavior, and increases mortality risk in older adults (3). Further, even though pain is one of the most common reasons adults seek medical care (4), it is often inadequately managed in older adults due to a multitude of factors (5). Therefore, identifying effective pain interventions and successful implementation of those interventions in the real word is essential for pain management in this population. The goal of this special edition Research Topic is to shed light on the progress made in the past decade in the geriatric pain field, with a focus on new insights, novel developments, current challenges, recent advances, and future perspectives in the field of geriatric pain. This editorial highlights the contributions of four papers that move the field forward in a diverse set of geriatric pain topics that hold particular relevance for the assessment and management of pain in frail older adults.

Older adults with chronic pain are more susceptible to becoming frail, characterized by an increased vulnerability to stressors and adverse health outcomes (6). Past research suggests the pain-frailty relationship is likely complex and not fully understood. In a novel study, Dong et al. explored the cross-sectional relationship between different pain characteristics (intensity, frequency, duration, and extent) and frailty in older adults (>75 years) at high risk of hospitalization. The data were collected as part of a larger pragmatic, multicenter trial at primary care practices in Sweden. In this vulnerable population of older adults, pain frequency was a stronger predictor of frailty compared to pain intensity, duration and extent (i.e., local vs. regional vs. widespread). However, when psychological factors and physical functioning were included in the prediction model, activities of daily living dependency had the strongest association with frailty. Longitudinal studies are needed to further explore whether pain frequency increases the risk of frailty in older adults at a high risk of hospitalization.

Because frailty is a common and debilitating condition in older adults with chronic pain, effective utilization of evidence-based pain treatments and assessments in this population is imperative. However, adopting and integrating pain interventions in clinical care settings comes with significant challenges. The article by Riffin et al. “*Program of all-inclusive care for the elderly: an untapped setting for research to advance pain care in older persons*” offers pain researchers a roadmap for collaborating with the Program of All-Inclusive Care for the Elderly (PACE) organizations to embed and assess evidenced-based pain interventions in older adults. PACE programs, offered in 32 states in the US, are designed to provide interdisciplinary care (i.e., physicians, nurses, physical therapists, social workers, etc.) to low-income older adults who qualify for nursing home admission but decide to obtain long-term care in community-settings. The authors draw on examples from their own multi-site clinical trial to provide practical recommendations for forging and sustaining research-PACE partnerships that could be applied to evidenced-based pain interventions. As described by the authors, research-PACE partnerships offer geriatric pain researchers' unique opportunities to study the transfer of empirically supported pain interventions and assessment into the real world for frail older adults.

Geriatric hip fractures have become a substantial health challenge worldwide, with a rising prevalence (7), an alarming postoperative mortality rate, and a high risk for postoperative health complications (8). Currently, the standard perioperative analgesia for hip fracture surgery is intravenous analgesia, primarily using opioids. Opioid use poses significant risk for adverse events in older adults, particularly in those who are frail. The article by Liu et al. presents a systematic review and meta-analysis for a promising treatment alternative, an ultrasound-guided fascia iliac compartment block (UG-FICB), to intravenous analgesia for acute pain management following hip fracture surgery in older adults. Importantly, the meta-analysis of 14 randomized controlled trials indicated that UG-FICB compared to intravenous analgesia significantly reduced pain up to 48 h post-surgery and reduced drug-related adverse events. These promising results, as well as the limitations of UG-FICB, are discussed in detail in the Liu article.

The article by Brooks et al. is a qualitative study that provides a description of the Mobile Intervention to Reduce Pain and Improve Health (MORPH) study and qualitatively explores participants views of the intervention. The MORPH pilot randomized controlled trial tests a hybrid remote and in-person physical activity intervention for obese, sedentary older adults with chronic pain. The trial takes a novel approach to activity promotion by utilizing a mobile health (mHealth) intervention and by focusing on the accumulation of steps throughout the day (vs. structured exercise), thereby interrupting prolonged periods of sitting. Overall, the authors detail the key lessons learned through the qualitative interviews, centering on the MORPH technology (e.g., Fitbit, MORPH Companion App), the intervention components, and weekly group meetings. These lessons can be used by other researchers to guide the development of future similar physical activity interventions to manage pain in obese older adults with chronic pain.

Collectively, this series of articles provide novel insights in geriatric pain that can be used to enhance the development, implementation, and testing of acute and chronic pain interventions in older adults.
